# Population-based analysis of non-steroidal anti-inflammatory drug use among children in four European countries in the SOS project: what size of data platforms and which study designs do we need to assess safety issues?

**DOI:** 10.1186/1471-2431-13-192

**Published:** 2013-11-19

**Authors:** Vera E Valkhoff, René Schade, Geert W ‘t Jong, Silvana Romio, Martijn J Schuemie, Andrea Arfe, Edeltraut Garbe, Ron Herings, Silvia Lucchi, Gino Picelli, Tania Schink, Huub Straatman, Marco Villa, Ernst J Kuipers, Miriam CJM Sturkenboom

**Affiliations:** 1 Department of Medical Informatics, Erasmus University Medical Center, Dr. Molewaterplein, Rotterdam, The Netherlands; 2 Department of Gastroenterology and Hepatology, Erasmus University Medical Center, Dr. Molewaterplein, Rotterdam, The Netherlands; 3 Department of Pediatrics, Sophia Children’s Hospital, Erasmus University Medical Center, Dr. Molewaterplein, Rotterdam, The Netherlands; 4 Division of Clinical Pharmacology & Toxicology, Hospital for Sick Children, University Avenue, Toronto, ON, Canada; 5 Division of Biostatistics and Public Health, Department of Quantitative Methods, University of Milano-Bicocca, Via Bicocca degli Arcimboldi, Milan, Italy; 6 Department of Clinical Epidemiology, Leibniz Institute for Prevention Research and Epidemiology-BIPS, Bremen, Germany; 7 PHARMO Institute, Van Deventerlaan, Utrecht, The Netherlands; 8 Local Health Authority ASL Cremona, Via San Sebastiano, Cremona, Italy; 9 International Pharmacoepidemiology and Pharmacoeconomics Research Center, Desio 20033, Italy; 10 Department of Epidemiology, Erasmus University Medical Center, Dr. Molewaterplein, Rotterdam, The Netherlands

**Keywords:** Pharmacoepidemiology, Database, Drug utilization, Health resource utilization, Drug safety, Sample size, Asthma exacerbation, Self-controlled case series design, Case-crossover design

## Abstract

**Background:**

Data on utilization patterns and safety of non-steroidal anti-inflammatory drugs (NSAIDs) in children are scarce. The purpose of this study was to investigate the utilization of NSAIDs among children in four European countries as part of the Safety Of non-Steroidal anti-inflammatory drugs (SOS) project.

**Methods:**

We used longitudinal patient data from seven databases (GePaRD, IPCI, OSSIFF, Pedianet, PHARMO, SISR, and THIN) to calculate prevalence rates of NSAID use among children (0–18 years of age) from Germany, Italy, Netherlands, and United Kingdom. All databases contained a representative population sample and recorded demographics, diagnoses, and drug prescriptions. Prevalence rates of NSAID use were stratified by age, sex, and calendar time. The person-time of NSAID exposure was calculated by using the duration of the prescription supply. We calculated incidence rates for serious adverse events of interest. For these adverse events of interest, sample size calculations were conducted (alpha = 0.05; 1-beta = 0.8) to determine the amount of NSAID exposure time that would be required for safety studies in children.

**Results:**

The source population comprised 7.7 million children with a total of 29.6 million person-years of observation. Of those, 1.3 million children were exposed to at least one of 45 NSAIDs during observation time. Overall prevalence rates of NSAID use in children differed across countries, ranging from 4.4 (Italy) to 197 (Germany) per 1000 person-years in 2007. For Germany, United Kingdom, and Italian pediatricians, we observed high rates of NSAID use among children aged one to four years. For all four countries, NSAID use increased with older age categories for children older than 11. In this analysis, only for ibuprofen (the most frequently used NSAID), enough exposure was available to detect a weak association (relative risk of 2) between exposure and asthma exacerbation (the most common serious adverse event of interest).

**Conclusions:**

Patterns of NSAID use in children were heterogeneous across four European countries. The SOS project platform captures data on more than 1.3 million children who were exposed to NSAIDs. Even larger data platforms and the use of advanced versions of case-only study designs may be needed to conclusively assess the safety of these drugs in children.

## Background

Non-steroidal anti-inflammatory drugs (NSAIDs) are frequently used for their analgesic, antipyretic, and anti-inflammatory effects, even in children. NSAIDs were the tenth most frequently prescribed drug in the age group 2–11 years (33 users/1000 person years) and the sixth most frequently prescribed drug in age group 12–18 years (57 users/1000 person years) in a combined primary care database study conducted in Italy, the Netherlands and the United Kingdom [1].

The Safety of Non-steroidal Anti-inflammatory Drugs (SOS) project is a research and development project funded by the Health Area of the European Commission under the Seventh Framework Programme, with the aim to assess the cardiovascular and gastrointestinal safety of NSAIDs, in particular with respect to children [2]. In the SOS project, prior to conducting novel observational studies on NSAID safety by linking seven databases from four European countries, data from published clinical trials and observational studies have been investigated by literature review and meta-analysis. This literature review revealed that safety of NSAIDs in children has not been adequately assessed in clinical trials nor post-marketing studies since most of these studies were too small and short to detect infrequent adverse events. In addition, the Paediatric Working Party of the European Medicines Agency (EMA) has identified the need to study safety issues related to specific NSAIDs, such as diclofenac, ibuprofen, ketoprofen, and naproxen [3].

In this study, as part of the SOS project, we aimed to investigate NSAID utilization patterns among children in four European countries and assess statistical power to study NSAID safety for ten adverse events of interest.

## Methods

### Data sources

Data for this study were obtained from seven longitudinal observational databases from four European countries involving medical data from more than 32 million people. Three primary care databases and four hospital discharge or administrative databases provided data from Germany (DE), Italy (IT), the Netherlands (NL) and the United Kingdom (UK) (Table 1). All databases recorded demographics, diagnoses, and drug prescriptions. Participating databases contain a representative sample of the respective populations based on age and sex. This analysis was exclusively based on routinely collected anonymized data and adhered to the European Commission’s Directive 95/46/EC for data protection. The protocol for this drug-utilization study was approved by the databases’ scientific and ethical advisory boards or regulatory agencies where applicable. The databases are described as follows.

**Table 1:** Study population and database characteristics

						**Pediatric source population**		
						**(Age 0 to 18 years)**		
**Database**	**Country**	**Type of database**	**Diagnoses captured with:**	**Drugs captured with:**	**Study period**	**Number of persons**	**Person-years of observation**	**Number of NSAID users**
GePaRD	Germany	Claims database	ICD-10-GM	ATC	2005 - 2008	2,992,087	7,056,919	925,667
THIN	United Kingdom	General practice database	READ	BNF/Multilex/ATC	1999 – 2008	1,261,668	5,198,351	227,927
IPCI	Netherlands	General practice database	ICPC and free text	ATC	1999 – 2011	250,296	618,479	12,002
PHARMO	Netherlands	Record linkage system	ICD-9-CM	ATC	1999 – 2008	594,800	2,914,576	82,233
OSSIFF	Italy	National Health Services registry (claims)	ICD-9-CM	ATC	2000 – 2008	675,197	3,671,014	22,760
SISR	Italy	National Health Services registry (claims)	ICD-9-CM	ATC	2002 – 2009	1,744,525	9,111,635	34,308
Pedianet*	Italy	General practice pediatric database	ICD-9-CM and free text	ATC	2000 – 2010	221,115	1,064,867	34,575
**Total**						**7,739,688**	**29,635,841**	**1,339,472**

*Pedianet only includes children up to the age of 14 years.

ICD-10-GM: International Classification of Diseases, 10th Revision German Modified; ICD-9-CM: International Classification of Diseases, 9th Revision Clinically Modified; ICPC: International Classification for Primary Care; ATC: Anatomical Therapeutic Chemical classification; BNF: British National Formulary.

#### German pharmacoepidemiological research database (GePaRD)

GePaRD is a claims database and consists of claims data from four German statutory health insurance (SHI) providers. It covers about 14 million persons throughout Germany who have at any time between 2004 and 2008 been enrolled in one of the four SHIs. The database population represents approximately 17% of the German population. Available data contain demographic information and information on hospital discharges, outpatient physician visits, and outpatient dispensing of prescribed medications in the pharmacies. Hospital diagnoses are coded according to the German Modification of the International Classification of Diseases, 10th Revision (ICD-10 GM) with at least 4 digits [4]. Information on drug prescriptions is linked to a pharmaceutical reference database providing information on the World Health Organization’s (WHO) anatomical-therapeutic-chemical (ATC) code [5], prescribed quantity (number of packages), prescription date, dispensation date, substance, product name, manufacturer, pack size, strength, defined daily dose (DDD), and pharmaceutical formulation. All involved SHIs, the Federal Ministry of Health (for data from multiple federal states) and the health authority of Bremen (for data from the Federal State of Bremen) approved the use of the data for this study.

#### The Health Improvement Network (THIN) database

THIN is a longitudinal database of primary care medical records from more than 10 million people in the UK. Some electronic records date back to 1985. Currently, the database has 3.6 million active patients registered. Data recorded in THIN include demographics, diagnoses, symptoms, prescriptions, life style information such as smoking or alcohol consumption, test results, height, weight, referrals to hospitals and specialists, and, on request, specialist letters and hospital discharge summaries. Diagnoses and symptoms are recorded using READ codes. Information on drug prescriptions is coded with MULTILEX product dictionary, mapped to ATC codes, and contains dose and duration. Approval for this study has been obtained from the Scientific Review Committee for the THIN database.

#### Integrated Primary Care Information (IPCI) database

The IPCI database is a dynamic longitudinal primary care research database from NL initiated in 1992. Currently, it covers about one million people from 150 active general practices. Symptoms and diagnoses are recorded using the International Classification for Primary Care (ICPC [6]) and free text and hospital discharge summaries. Information on drug prescriptions comprises official label text, quantity, strength, prescribed daily dose and is coded according to the ATC classification. Approval for this study has been obtained from the IPCI-specific ethical review board ‘Raad van Toezicht’.

#### PHARMO database

The PHARMO medical record linkage system is a population-based patient-centric data tracking system of 3.2 million community-dwelling inhabitants from NL. Data have been collected since October 1994. The drug dispensing data originate from out-patient-pharmacies. Via the Dutch National Medical Register (LMR) hospital admissions are collected with ICD-9-clinically modified (CM). Information on drug prescriptions is coded according to the ATC classification.

#### Osservatorio Interaziendale per la Farmacoepidemiologia e la Farmacoeconomia (OSSIFF) database

In the Italian National Health Service (NHS), the Local Health Authority is responsible for the health of the citizens in a given geographical area, usually a province. In 2006, eight authorities have established a network named OSSIFF, accounting for a population of about 3.8 million people. Hospital diagnoses are coded according to ICD-9-CM. Prescriptions are coded according to the ATC coding system, and additionally prescription date, number of prescribed units, drug strength and the defined daily dose (DDDs) of the active entity are available.

#### Sistema Informativo Sanitario Regionale (SISR) database

In the Italian SISR database, data are obtained from the electronic healthcare databases of the Lombardy region. Lombardy is the largest Italian region with about nine million inhabitants, about 16% of the population of Italy. This population is entirely covered by a system of electronically linkable databases containing information on health services reimbursable by the NHS. The SISR database has complete population coverage and data is available from 2002. Via the ICD-9-CM dictionary and ATC classification, the database captures information on diagnoses from hospitalizations and drugs. Because OSSIFF covers a subset of patients covered by SISR, this database excluded the common subset of patients to avoid overlap.

#### Pedianet database

The Italian Pedianet database is a primary care pediatric database comprising the clinical data of about 160 family pediatricians (FPs) distributed throughout Italy. In Italy all children until the age of 14 years are registered with an FP. Pedianet has been built up since 1999. By December 2010, Pedianet database contained data on 370,000 children. Information on all drugs (date of prescription, ATC code, substance, formulation, quantity, dosing regimen, legend duration, indication, reimbursement status), symptoms and diagnoses are available in free text or coded by the ICD-9 system.

### Data sharing and data extraction

In accordance with European data protection standards, neither personal identifiers nor other patient-level data were shared across countries. Data were extracted and processed locally by Jerboa© software, a software developed and validated at Erasmus University Medical Center in Rotterdam [7]. The Jerboa software calculated drug-utilization and disease-incidence measures for each database stratified by age, sex, and calendar time. The concept of a distributed data network with a common format of input files has been described previously [7]. The aggregated and de-identified data were stored centrally at a data warehouse (DW) in Milan, Italy. Assigned persons were allowed to gain access to the DW via a secured token, assigned to an Internet Protocol (IP)-address.

Three input files were extracted from each database locally according to a pre-specified common format containing information on: (i) patient characteristics such as date of birth, sex, and registration date; (ii) NSAID prescriptions or dispensing (ATC code M01A) including duration of supply, and (iii) diagnoses and their corresponding date through ICD-10, READ, ICD-9, ICPC codes or free text. The observation time for each patient started 365 days after registration with a practice or health insurance system. For children who were born into the database, observation started at date of birth. The observation period ended at the earliest of the following dates: turning 14 (Pedianet) or 18 years of age, transfer out of the practice or insurance system, death, or last data collection. The study period varied between databases according to data availability (Table 1).

### Events of interest for safety assessment

The pediatric part of the SOS project considered the following ten outcomes that are of clinical relevance in children: asthma exacerbation, anaphylactic shock, upper gastrointestinal complications, stroke, heart failure, acute renal injury, Stevens–Johnson syndrome, acute liver injury, acute myocardial infarction, and Reye’s syndrome [8-16].

To extract the events of interest in the participating databases, the medical concepts were first mapped using the Unified Medical Language System (UMLS), a biomedical terminology integration system handling more than 150 medical dictionaries [17]. This process was needed as the clinical information captured by the different databases is collected using four different disease terminologies (ICPC, ICD-9, ICD-10, and READ codes) and free text in Dutch and Italian. For each medical concept, UMLS identified corresponding codes for each of the four terminologies. This UMLS-based approach was developed in the EU-ADR project and has been described in more detail elsewhere [18]. Subsequently, the codes were extracted in a centralized process (referred to as the codex method) and reviewed by a panel of medically trained investigators according to event definitions. Extraction queries were reviewed in case of large, unexpected discrepancies. This harmonization process enabled a more homogeneous identification of events across databases using different coding-based algorithms.

### Statistical analyses

#### Drug utilization measures

For each database, the prevalence rate of NSAID use was calculated by dividing the number of prevalent NSAID users by the person-time of observation, stratified by age, sex, calendar year, and calendar month. The reference calendar year was 2007. The person-time of NSAID exposure was calculated by using the duration of the prescription supply. Relative prevalence rates (in percentages) were calculated by dividing the absolute prevalence rate by the mean prevalence rate within each database for each calendar month and one-year age category.

#### Incidence rates for events of interest

We calculated incidence rates (IRs) per 100,000 person-years for each of the events of interest for each database and performed direct standardization using the WHO World Standard Population as reference to account for age differences when comparing the overall diagnosis rates (standardized IRs; SIRs) [19]. We only considered the first recorded occurrence of the event of interest after a run-in period of one year. To calculate the overall IR in the SOS platform, the total number of events across databases was divided by the person time captured in all databases.

#### Required amount of drug exposure to detect safety signals

To determine the usability of the SOS database platform for the study of NSAID safety with respect to adverse events of interest in children, we calculated the person-years of exposure required to detect a drug-event association over varying magnitudes of relative risks (RR), using RRs of 2 (weak association), 4 (moderate association), and 6 (strong association), a one-sided significance level (α) of 0.05, and a power (1-β) of 80%. To estimate the required exposure for specific strengths of association we used a previously published sample size formula [20]. The required exposure time was compared to the person time of exposure to ibuprofen to assess whether the database platform is sufficient in current size, or expansion would be necessary for adequate evaluation of safety.

## Results

### Source population

The pediatric population of the SOS platform network comprised 7.7 million children and adolescents (0 to 18 years) contributing 29.6 million person-years (PYs) of observation between 1999 and 2011 (Table 1). Of the observation time, 11.5% were for children less than 2 years of age, 20.8% for children aged 2 to ≤5 years, 31.5% for children aged 6 to ≤11 years and 36.3% for adolescents aged 12 to ≤18 years. Of the combined pediatric population, 51.4% were male. The database which contributed most person time was SISR, followed by GePaRD and THIN, with different observation periods across databases according to data availability (Table 1).

### Prevalence of NSAID use

Of the 7.7 million children and adolescents, 1,339,472 (17.3%) used one of the 45 NSAIDs for at least one day during observation time (Table 1). This generated a total exposure of 61,739 PYs of NSAID exposure. In GePaRD, 31% of children used NSAIDs, which is in contrast with lower percentages in SISR (2%), OSSIFF (3%), and IPCI (5%).

**Figure 1:**
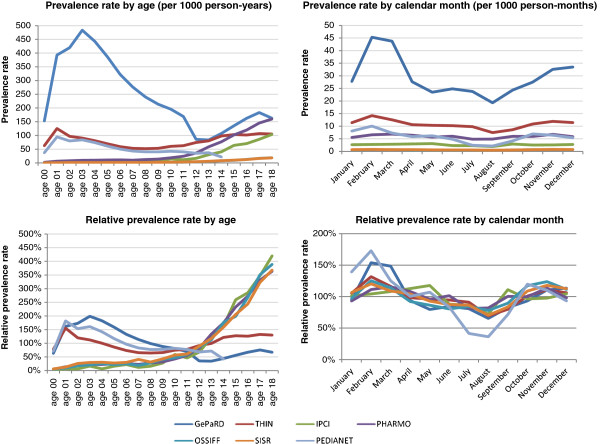
Prevalence rates (top) and relative prevalence rates (bottom) of NSAID use for the calendar year 2007, for each database, by age (left) and by calendar month (right).

**Figure 2:**
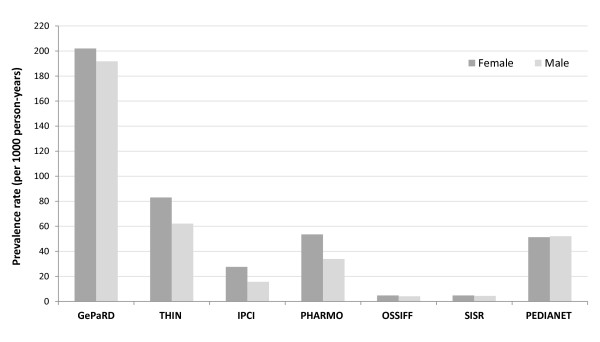
Prevalence rates of NSAID use for the calendar year 2007, for each database, stratified by sex.

The overall prevalence rate of NSAID use was 56 per 1,000 person-years in 2007, and ranged between 4.4 in OSSIFF and 197 in GePaRD. Figure 1 shows that the annual prevalence of NSAID use varies between age groups and countries. There were two distinct prescription patterns. The first pattern showed that the prevalence of NSAID use was relatively low in young children and substantially higher for children older than 8 years of age for IPCI, PHARMO, OSSIFF and SISR. In contrast, the use of NSAIDs was most prevalent before the age of four in children for GePaRD, THIN and Pedianet. In GePaRD, prevalence rates reached values of 483 per 1000 PYs (48% of children) for three-year-olds in 2007. Prevalence rates decreased and were lowest for the age categories of thirteen and eight years for GePaRD and THIN, respectively. The prevalence rates of NSAID use increased thereafter. Figure 2 shows that the overall annual prevalence rates of NSAID use in 2007 were higher for females than for males, especially for THIN, IPCI and PHARMO. The sex distribution was equal for all databases until the age of ten, but the prevalence rates diverge after that age with higher rates for females in GePaRD, THIN, IPCI and PHARMO. Annual prevalence of NSAID use was relatively stable over calendar time for most databases. There was a tendency of slightly decreasing prevalence rates after the year 2003 for OSSIF and SISR while prevalence rates were steadily increasing for THIN and GePaRD (data not shown).

Monthly prevalence rates of NSAID use showed that prescriptions were most common in February and less frequent in summer months. This seasonal pattern of NSAID use in children and adolescents was especially seen in GePaRD (August: 19; February: 45), THIN (August: 7.5; February: 14), and Pedianet (August: 2.1; February: 10 – all numbers per 1000 person months in 2007) (Figure 1). Mean duration of NSAID prescription or dispensing was highest in THIN and SISR (15.4 and 15.8 days) and lowest in Pedianet (4.8 days).

### Individual NSAIDs

On average, 26 NSAIDs were prescribed or dispensed per database with a range between 19 for IPCI and 32 for OSSIFF. Of those, ibuprofen was the most frequently used NSAID, accounting for 69.3% of total person time of NSAID exposure. Diclofenac and naproxen were also available in all databases and accounted for 13.0% and 6.3% of the total person time of NSAID exposure, respectively. Distribution of NSAID use was heterogeneous between countries. Ibuprofen was the most frequently used NSAID in GePaRD, THIN and Pedianet, while nimesulide was most frequent in the other two Italian databases (OSSIFF and SISR), followed by ketoprofen and naproxen. Together with ibuprofen and ketoprofen, morniflumate was common in Pedianet. In the Netherlands (IPCI and PHARMO), diclofenac, naproxen and ibuprofen were most common. Nimesulide, morniflumate and niflumic acid were only available in Italy, while lonazolac and parecoxib were only available in the GePaRD database (Germany), and etodolac, fenbufen, and fenoprofen were only prescribed in THIN (UK). In IPCI and PHARMO (both from NL) a fixed combination of diclofenac and misoprostol (a prostaglandin E1 analogue used for gastroprotection) was frequently prescribed to adolescents, whereas this was not common in other databases (data not shown). In all databases except OSSIFF and SISR (both from IT), the three most frequently used NSAIDs accounted for more than 80% of the total person-years of NSAID exposure. Proprionic acid derivates (such as ibuprofen; ATC code M01AE) were by far most common in all databases except OSSIFF and SIRS. OSSIFF and SIRS showed highest prescription rates for cyclooxygenase-2-selective NSAIDs (coxibs; 12% and 8.3% respectively, as compared to an average of 1.2% for the other database).

### Required exposure time for NSAID safety assessment in children

Table 2 shows the number of NSAIDs that have enough exposure to detect weak (RR = 2), moderate (RR = 4) or strong (RR = 6) associations for the ten adverse events of interest. The stronger the association and the more common the event to be studied, the lower is the required exposure time for a specific NSAID substance. Thus, the lower the required exposure time for a specific NSAID substance the higher is the number of drugs that can be studied, which is expected from the power calculations. Taking asthma exacerbation as example with the highest incidence rate (IR) of 82/100 000 PYs, only one NSAID (ibuprofen) had enough person time exposure (9,788 person-years or more) to detect a weak association (RR = 2). To assess a moderate (RR = 4) or a strong (RR = 6) association with asthma exacerbation, four and six NSAID substances had adequate person time of exposure, respectively. None of the drugs accounted for adequate exposure time to detect a strong association for the following rare events: Stevens-Johnson syndrome, acute liver failure, acute myocardial infarction, and Reye’s syndrome. For a very rare outcome such as Reye’s Syndrome, the SOS platform would require 998 times as much exposed person time in order to study a weak association for ibuprofen (the most commonly used NSAID) (Table 2). Table 3 shows for which events of interest sufficient person time was available to study a strong association (RR = 6) for the most frequently used NSAIDs.

**Table 2:** Required exposure time needed to investigate NSAID safety in children for ten potential adverse events with varying incidence rates considering a weak, moderate or strong association

**Event type**	**IR/100,000 PY**	**Weak association**			**Moderate association**			**Strong association**		
		**(RR = 2)**			**(RR = 4)**			**(RR = 6)**		
		**Required exposure (PY)**	**Drugs**	**Expan-sion**	**Required exposure (PY)**	**Drugs**	**Expan-sion**	**Required exposure (PY)**	**Drugs**	**Expan-sion**
			**N (%)**			**N (%)**			**N (%)**	
Asthma exacerbation	82.12	9,788	1 (2.2)	0	1,499	4 (8.9)	0	669	6 (13.3)	0
Anaphylactic shock	4.29	187,358	0 (0)	4	28,687	1 (2.2)	1	12,809	1 (2.2)	0
Upper gastrointestinal complication	2.64	303,990	0 (0)	7	46,545	0 (0)	1	20,782	1 (2.2)	0
Stroke	2.07	388,410	0 (0)	9	59,471	0 (0)	1	26,554	1 (2.2)	1
Heart failure	1.57	511,927	0 (0)	12	78,384	0 (0)	2	34,998	1 (2.2)	1
Acute renal failure	1.40	573,919	0 (0)	13	87,875	0 (0)	2	39,236	1 (2.2)	1
Stevens–Johnson syndrome	0.56	1,438,097	0 (0)	34	220,194	0 (0)	5	98,315	0 (0)	2
Acute liver failure	0.46	1,741,369	0 (0)	41	266,629	0 (0)	6	119,048	0 (0)	3
Acute myocardial infarction	0.12	6,918,411	0 (0)	162	1,059,310	0 (0)	25	472,974	0 (0)	11
Reye’s syndrome	0.02	42,663,537	0 (0)	998	6,532,413	0 (0)	153	2,916,676	0 (0)	68

IR: incidence rate; RR: relative risk; PY: Person years.

Drugs N (%): Number of drugs that have enough PY of exposure in the SOS platform to detect a potential signal for the respective event of interest (in brackets the proportion of NSAIDs with enough PY exposure of all 45 NSAIDs).

Expansion: magnitude of enlargement of PY exposure in the SOS platform necessary for assessment of each safety outcome for ibuprofen (exposed person time 42,768 PY) given the specified relative risk that should be detected with α<0.05 (one-sided) and ß = 0.20.

**Table 3:** Is sufficient exposure time available in the SOS platform to investigate the particular event of interest given an expected relative risk of six stratified by NSAID substance?

**Given an RR of 6:**												
**ATC**	**SUM PYs**	**% PYs**	**Asthma exacerbation**	**Anaphylactic shock**	**Upper gastrointestinal complication**	**Stroke**	**Heart failure**	**Acute renal failure**	**Stevens–Johnson syndrome**	**Acute liver failure**	**Acute myocardial infarction**	**Reye’s syndrome**
**Total NSAIDs**	**61,739**	**100**	**X**	**X**	**X**	**X**	**X**	**X**				
Ibuprofen^*^	42,768	69.3	**X**	**X**	**X**	**X**	**X**	**X**				
Non-ibuprofen^+^	18,971	30.7	**X**	**X**	**(X)**							
Diclofenac^#^	8,000	13.0	**X**									
Naproxen^^^	3,878	6.3	**X**									
Mefenamic acid	2,297	3.7	**X**									
Ketoprofen^&^	946	1.5	**X**									
Nimesulide	925	1.5	**X**									
Piroxicam	519	0.8										
Indometacin	440	0.7										
Meloxicam	328	0.5										
Celecoxib	258	0.4										
Rofecoxib	247	0.4										
Etoricoxib	218	0.4										

X: denotes that enough person time is available for detection of a RR of 6 with α = 0.05 (one-sided) and ß = 0.20; (X): denotes that enough person time is available for detection of a RR of 6 with α = 0.1 (one-sided) and ß = 0.20, exclusive to the use of α = 0.05; PYs: denotes Person years.

^+^including all NSAID preparation without ibuprofen.

^*^including combinations with ibuprofen.

^#^including combinations with diclofenac.

^^^including combinations with naproxen.

^&^including combinations with ketoprofen.

## Discussion

In the SOS project, the combined source population of children and adolescents (0 to 18 years of age) from seven databases from four European countries involved 7.7 million children and adolescents and generated 29.6 million person-years of observation between 1999 and 2011. Of these, 1.3 million children received NSAID prescriptions during the studied periods in the respective databases. Overall, 56 children/adolescents out of 1000 received an NSAID prescription per year. This varied largely between 4 per 1000 in OSSIFF to 197 per 1000 in GePaRD in the pediatric population. In general, one could conclude that the annual prevalence of prescribed NSAIDs is lowest in Italy, followed by the Netherlands, the United Kingdom and highest for Germany. Also, in all databases except the Italian ones, females received more NSAID prescriptions than males, mainly related to diverging prevalence rates in adolescence (Figure 2). When considering the age-specific prevalence rates, the high rates in the very young for the German database GePaRD compared to the other European countries are striking (Figure 1). For GePaRD values reach prevalence rates greater than 480 (48% of children in one year) for 3-year-olds. In Germany, United Kingdom and Italy, ibuprofen is the drug of choice beside paracetamol (acetaminophen) for fever in children [21-23], whereas in the Netherlands paracetamol is considered first [24]. In THIN and Pedianet prevalence rates were also higher in children below the age of 4, whereas for other databases prevalence rates were steadily increasing with age and peak at the age of 18. In the same three databases with high NSAID use in young children a clear seasonality is seen with highest NSAID use in winter, probably related to prescription of NSAIDs to young children for fever and fever-like symptoms (Figure 1). Between countries major differences exist in the type of NSAID that was used. Ibuprofen was the most frequently used NSAID (69.3%). Safety and efficiency of ibuprofen in children are much more extensively studied than (most) other NSAIDs [10-13].

Two databases from the Netherlands were included in this study, allowing a comparison between populations that should have similar characteristics. Since PHARMO is a pharmacy dispensing database that captures over-the-counter (OTC) dispensations of NSAIDs, the prevalence of NSAID exposure was slightly higher for PHARMO than for IPCI, especially in adolescents. Three Italian databases participated in the SOS platform and the prevalence rates for different ages of NSAID use were very similar for OSSIFF and SISR, but not for Pedianet (Figure 1). This could be related to the fact that Pedianet captures all prescriptions, whether reimbursed or not, plus recommendations on NSAID treatment made by pediatricians, while OSSIFF and SISR contain only the reimbursed NSAID dispensing.

Although the SOS platform appears to provide a unique opportunity to study the safety of NSAIDs in a large number of children and adolescents, we showed that the data are still too limited to study the safety of specific NSAID substances or the safety of NSAIDs in general for rare adverse drug reactions. Only for ibuprofen enough exposure time was available in the platform to investigate the risk of asthma exacerbation (the most common event) for a ‘weak association’ with a RR of 2. Data accumulation in platforms like SOS and others is of utmost importance for the safety evaluation of drugs in adults and children. The coming decade is likely to bring enormous expansion of available health care records, and advancement of data mining and harmonisation methods. Both the U.S. Food and Drug Administration and the European Medicines Agency invest in infrastructure and knowledge expansion in this field. However, our study shows how difficult it is to study safety in children, when compared to adults. Because of lower drug consumption – fortunately – use of these platforms for adequate drug safety surveillance is more challenging, as are many aspects of drug research in children. This should emphasize the responsibility as researchers, clinicians, and policy makers to facilitate high quality research in this vulnerable patient group through funding, scholarship, education and collaboration.

### Limitations

Some limitations should be considered. First, in this analysis, we primarily used alpha = 0.05 as a testing threshold. To propose a tentative signal for NSAID safety in the pediatric population, a less stringent testing threshold may be indicated. For an expected RR of 6, a ‘strong association’ , we performed additional power calculations with a less stringent alpha value of 0.1 (Table 3). This sensitivity analysis did not materially change our results. Second, our study may not have captured all NSAID exposure, since many of these drugs are also available without prescription in all four countries. We expect any underestimation of NSAID use in the present study to be minor since most parents may be reluctant to administer drugs to their children without having consulted a health care professional. In addition, people are likely to prefer prescribed over freely available NSAIDs for financial reasons since reimbursement is only possible for prescribed drugs. Third, we observed that rates of NSAID use were low in the month of August. This is to be expected because of summer holiday periods during which physician or pharmacy visits are less likely to occur. Fourth, we only used diagnosis codes for identification of pediatric events of interest. We did neither use laboratory values, medical images nor procedures for event measurement, therefore potentially missing some events. We expect the amount of misclassification to be very minor since most patients with a confirmed diagnosis from these examinations would have a diagnosis code entered in the participating databases, as this is important for reimbursement. Fifth, we only considered the total person time of NSAID exposure, thereby possibly overestimating the possibilities of safety assessment. Issues such as gap lengths between subsequent NSAID prescriptions and switching between different substances would have to be accounted for by design of NSAID safety studies. Biases related to prevalent NSAID users can be avoided with a new-user study design [25]. With a new-user design, however, prevalent NSAID users would be excluded from the study cohort, thereby resulting in less exposure time than presented in this analysis.

For the SOS studies, to estimate outcome risks with NSAID use in children and adolescents, we will consider case-only designs such as self-controlled case series or case-crossover [26]. One advantage is that case-only designs automatically control for all time-invariant confounders, measured or unmeasured (e.g., gender or genetics). They also produce better estimates in terms of statistical power to detect a safety signal when compared with cohort studies or case–control studies, thus offering a possibility to overcome limited data resources such as in the present context [27]. For several pediatric outcomes of interest, the occurrence of the event may change the probability of subsequent NSAID exposure, either by contraindication (e.g., acute renal failure and anaphylactic shock) or increased mortality risk (e.g., acute myocardial infarction and stroke), thereby violating the event-independent exposure assumption of the standard self-controlled case series method [28]. These issues can be addressed with case-only designs by use of either an advanced version of the self-controlled case series method [29-31] or a case-crossover design [32]. The case-crossover design considers only pre-event time and can be extended by methods such as the case-time-control design to account for time trends of drug exposure [33,34].

## Conclusions

NSAID use is common in children and utilization patterns varied between Germany, Italy, United Kingdom, and The Netherlands. There is a clear need to study NSAID safety in children [3]. Although the SOS platform captures information on a large number of young NSAID users (1.3 million), even larger data platforms may be needed to conclusively assess the safety of these drugs in children, especially for rare events. International collaboration is needed to adequately study NSAID safety in children. Advanced versions of case-only study designs may be indicated to gain statistical power to study NSAID safety in children.

## Abbreviations

ATC: Anatomical therapeutic chemical classification system; BNF: British national formulary; CV: Cardiovascular; DDD: Defined daily dose; DW: Data warehouse; EMA: European medicines agency; EU: European union; FDA: U.S. Food and drug administration; FP: Family pediatrician/Office-based pediatrician; GE: Germany; GePaRD: German pharmacoepidemiological research database; GP: General practitioner/family physician; ICH: International conference of harmonization; IPCI: Integrated primary care information project; IT: Italy; NL: The Netherlands; NSAID: Non-steroidal anti-inflammatory drug; OSSIFF: Osservatorio Interaziendale per la Farmacoepidemiologia e la Farmacoeconomia; PY: Person-years (a commonly used denominator correcting for incomplete participation of individual patients); SAE: Serious adverse event; SISR: Sistema informativo sanitario regionale (Regional Health Informative System); THIN: The health improvement network; UK: United Kingdom; WHO: World health organization of the united nations (UN).

## Competing interests

Vera Valkhoff, as employee of Erasmus MC, has conducted research for AstraZeneca.

René Schade has no conflicts of interest to disclose.

Geert ’t Jong has no conflicts of interest to disclose.

Geert ‘t Jong had full access to all the data in the study and takes responsibility for the integrity of the data and the accuracy of the data analysis.

Silvana Romio has no conflicts of interest to disclose.

Martijn J. Schuemie has no conflicts of interest to disclose.

Andrea Arfe has no conflicts of interest to disclose.

Edeltraut Garbe runs a department that occasionally performs studies for pharmaceutical industries with the full freedom to publish. The companies include Mundipharma, Bayer, Stada, Sanofi-Aventis, Sanofi-Pasteur, Novartis, Celgene, and GSK. She has been consultant to Bayer-Schering, Nycomed, Teva, and Novartis in the past. The present work is unrelated to the above grants and relationships.

Ron Herings has no conflicts of interest to disclose.

Silvia Lucchi has no conflicts of interest to disclose.

Gino Picelli has conducted studies for Merck and Pfizer.

Tania Schink has no conflicts of interest to disclose.

Huub Straatman has no conflicts of interest to disclose.

Marco Villa has no conflicts of interest to disclose.

Ernst J Kuipers has no conflicts of interest to disclose.

Miriam Sturkenboom is head of a unit that conducts some research for pharmaceutical companies: Pfizer, Lilly and Altana.

## Authors’ contributions

VEV, RS, SR, and MCJMS participated in the conception and design of the study. VEV, RS, AA, EG, RH, SL, GP, TS, HS, MV, and MCJMS participated in the acquisition of data. VEV, RS, GW’tJ, SR, MJS, SL, EJK, and MCJMS participated in the analysis and interpretation of data. VEV, RS, and GW’tJ drafted the manuscript. All authors revised the manuscript for important intellectual content and approved the final manuscript.

## Pre-publication history

The pre-publication history for this paper can be accessed here:

http://www.biomedcentral.com/1471-2431/13/192/prepub

## References

[B1] Sturkenboom MC Verhamme KM Nicolosi A Murray ML Neubert A Caudri D Picelli G Sen EF Giaquinto C Cantarutti L Drug use in children: cohort study in three European countries BMJ 2008 337 a2245 10.1136/bmj.a2245 19029175 PMC2593449

[B2] Salvo F Fourrier-Reglat A Bazin F Robinson P Riera-Guardia N Haag M Caputi AP Moore N Sturkenboom MC Pariente A Cardiovascular and gastrointestinal safety of NSAIDs: a systematic review of meta-analyses of randomized clinical trials Clin Pharmacol Ther 2011 89 855 866 10.1038/clpt.2011.45 21471964

[B3] Assessment of the paediatric needs, pain. Paediatric working party of the european medicines agency 2005 Available at: http://www.ema.europa.eu/docs/en_GB/document_library/Other/2009/10/WC500004037.pdf (accessed April 4, 2012)

[B4] World Health Organization Classification of Diseases Available at: http://www.who.int/classifications/icd/en/. (accessed April 4, 2011)

[B5] WHO collaborating centre for drug statistics methodology. Guidelines for ATC classification and DDD assignment Available at: http://www.whocc.no/atcddd/. (accessed April 4, 2012)

[B6] Lamberts H Wood M Hofmans-Okkes IM International primary care classifications: the effect of fifteen years of evolution Fam Pract 1992 9 330 339 10.1093/fampra/9.3.330 1459391

[B7] Coloma PM Schuemie MJ Trifiro G Gini R Herings R Hippisley-Cox J Mazzaglia G Giaquinto C Corrao G Pedersen L Combining electronic healthcare databases in Europe to allow for large-scale drug safety monitoring: the EU-ADR Project Pharmacoepidemiol Drug Saf 2011 20 1 11 10.1002/pds.2053 21182150

[B8] Ashraf E Ford L Geetha R Cooper S Safety profile of ibuprofen suspension in young children Inflammopharmacology 1999 7 219 225 10.1007/s10787-999-0005-0 17638093

[B9] Lesko SM Louik C Vezina RM Mitchell AA Asthma morbidity after the short-term use of ibuprofen in children Pediatrics 2002 109 E20 10.1542/peds.109.2.e20 11826230

[B10] Lesko SM Mitchell AA An assessment of the safety of pediatric ibuprofen. A practitioner-based randomized clinical trial JAMA 1995 273 929 933 10.1001/jama.1995.03520360043037 7884951

[B11] Lesko SM Mitchell AA Renal function after short-term ibuprofen use in infants and children Pediatrics 1997 100 954 957 10.1542/peds.100.6.954 9374563

[B12] Lesko SM Mitchell AA The safety of acetaminophen and ibuprofen among children younger than two years old Pediatrics 1999 104 e39 10.1542/peds.104.4.e39 10506264

[B13] Pierce CA Voss B Efficacy and safety of ibuprofen and acetaminophen in children and adults: a meta-analysis and qualitative review Ann Pharmacother 2010 44 489 506 10.1345/aph.1M332 20150507

[B14] Southey ER Soares-Weiser K Kleijnen J Systematic review and meta-analysis of the clinical safety and tolerability of ibuprofen compared with paracetamol in paediatric pain and fever Curr Med Res Opin 2009 25 2207 2222 10.1185/03007990903116255 19606950

[B15] Sullivan JE Farrar HC Fever and antipyretic use in children Pediatrics 2011 127 580 587 10.1542/peds.2010-3852 21357332

[B16] Ulinski T Guigonis V Dunan O Bensman A Acute renal failure after treatment with non-steroidal anti-inflammatory drugs Eur J Pediatr 2004 163 148 150 10.1007/s00431-003-1392-7 14745553

[B17] Unified Medical Language System® (UMLS®) from the U.S. National Library of Medicine Available at: http://www.nlm.nih.gov/research/umls/. (accessed April 4, 2011)

[B18] Avillach P Mougin F Joubert M Thiessard F Pariente A Dufour JC Trifiro G Polimeni G Catania MA Giaquinto C A semantic approach for the homogeneous identification of events in eight patient databases: a contribution to the European eu-ADR project Stud Health Technol Inform 2009 150 190 194 19745295

[B19] World Health Organization WHO standard population 2001 Available at: http://www.who.int/healthinfo/paper31.pdf. (accessed April 4, 2012)

[B20] Coloma PM Trifiro G Schuemie MJ Gini R Herings R Hippisley-Cox J Mazzaglia G Picelli G Corrao G Pedersen L Electronic healthcare databases for active drug safety surveillance: is there enough leverage? Pharmacoepidemiol Drug Saf 2012 21 611 621 10.1002/pds.3197 22315152

[B21] National Collaborating Centre for Women’s and Children’s Health Feverish illness in children. Assessment and initial management in children younger than 5 years 2007 London: National Institute for Health and Clinical Excellence

[B22] Kindern F b Patientenleitlinien.de. Universität Witten-Herdecke 2006 Available at: http://www.patientenleitlinien.de/Fieber_Kindesalter/fieber_kindesalter.html (accessed April 4, 2012)

[B23] Chiappini E Venturini E Principi N Longhi R Tovo PA Becherucci P Bonsignori F Esposito S Festini F Galli L Update of the 2009 Italian pediatric society guidelines about management of fever in children Clin Ther 2012 34 1648 1653 e1643 10.1016/j.clinthera.2012.06.011 22742886

[B24] Berger M Boomsma L Albeda F Dijkstra R Graafmans T Van der Laan J Lemmen W Oteman N NHG-Standaard Kinderen met koorts (Tweede herziening) Huisarts Wet 2008 51 287 296 10.1007/BF03086785

[B25] Ray WA Evaluating medication effects outside of clinical trials: new-user designs Am J Epidemiol 2003 158 915 920 10.1093/aje/kwg231 14585769

[B26] Maclure M Fireman B Nelson JC Hua W Shoaibi A Paredes A Madigan D When should case-only designs be used for safety monitoring of medical products? Pharmacoepidemiol Drug Saf 2012 21 Suppl 1 50 61 22262593 10.1002/pds.2330

[B27] Farrington CP Nash J Miller E Case series analysis of adverse reactions to vaccines: a comparative evaluation Am J Epidemiol 1996 143 1165 1173 10.1093/oxfordjournals.aje.a008695 8633607

[B28] Farrington CP Relative incidence estimation from case series for vaccine safety evaluation Biometrics 1995 51 228 235 10.2307/2533328 7766778

[B29] Whitaker HJ Hocine MN Farrington CP The methodology of self-controlled case series studies Stat Methods Med Res 2009 18 7 26 10.1177/0962280208092342 18562396

[B30] Farrington CP Whitaker HJ Hocine MN Case series analysis for censored, perturbed, or curtailed post-event exposures Biostatistics 2009 10 3 16 18499654 10.1093/biostatistics/kxn013

[B31] Farrington CP Anaya-Izquierdo K Whitaker H Hocine MN Douglas I Smeeth L Self-controlled case series analysis with event-dependent observation periods J Am Stat Assoc 2011 106 494 417 426 10.1198/jasa.2011.ap10108

[B32] Maclure M The case-crossover design: a method for studying transient effects on the risk of acute events Am J Epidemiol 1991 133 144 153 1985444 10.1093/oxfordjournals.aje.a115853

[B33] Suissa S The case-time-control design Epidemiology 1995 6 248 253 10.1097/00001648-199505000-00010 7619931

[B34] Wang S Linkletter C Maclure M Dore D Mor V Buka S Wellenius GA Future cases as present controls to adjust for exposure trend bias in case-only studies Epidemiology 2011 22 568 574 10.1097/EDE.0b013e31821d09cd 21577117 PMC3110688

